# Effects of Exogenous SA/GABA Combined with ZnSO_4_ Treatment on the Physiological Metabolism and Flavonoid Biosynthesis in Finger Millet (*Eleusine coracana* L.) Sprouts

**DOI:** 10.3390/plants15132065

**Published:** 2026-07-02

**Authors:** Qianqian Zhu, Jing Zhang, Zhangqin Ye, Weiming Fang, Yongqi Yin

**Affiliations:** College of Food Science and Engineering, Yangzhou University, Yangzhou 225009, China; mx120251367@stu.yzu.edu.cn (Q.Z.); mz120222090@stu.yzu.edu.cn (J.Z.); mz120242240@stu.yzu.edu.cn (Z.Y.); wmfang@yzu.edu.cn (W.F.)

**Keywords:** flavonoid biosynthesis, salicylic acid, γ-aminobutyric acid, zinc sulfate

## Abstract

Finger millet (*Eleusine coracana* L.) is rich in bioactive compounds, including flavonoids. Following exogenous substance regulation, its sprouts can achieve efficient flavonoid enrichment. This study investigates the regulatory effects of exogenous salicylic acid (SA) and γ-aminobutyric acid (GABA) on the physiological metabolism, oxidative stress response, and flavonoid biosynthesis of finger millet sprouts subjected to 5 mM zinc sulfate (ZnSO_4_) stress. Compared to treatment solely with ZnSO_4_, the application of both 50 μM salicylic acid (SA) and 1 mM gamma-aminobutyric acid (GABA) markedly enhanced flavonoid biosynthesis, with respective yields of 8.53 μg/sprout and 8.85 μg/sprout observed by 6 days post-germination. Concurrently, SA and GABA attenuated ZnSO_4_-induced oxidative damage. During days 4 and 6 post-germination, malondialdehyde and hydrogen peroxide levels in sprouts were significantly reduced, with levels at 6 days showing a particularly notable decrease. Moreover, the catalytic activities of catalase, peroxidase, superoxide dismutase, and ascorbate peroxidase were significantly upregulated. Further analysis revealed that both treatments significantly activated the phenylpropanoid biosynthesis pathway. The activities of key rate-limiting enzymes, phenylalanine ammonia-lyase, cinnamate-4-hydroxylase, and 4-coumarate-CoA ligase, along with the expression levels of their corresponding genes, were markedly upregulated. Concurrently, the expression of genes and transcription factors, specifically myeloblastosis and NAC transcription factors, involved in regulating reactive oxygen species homeostasis also increased. These findings suggest that exogenous SA, GABA, and ZnSO_4_ cotreatment can effectively enhance the accumulation of flavonoids and the nutritional quality of finger millet sprouts by bolstering antioxidant capacity and modulating the flavonoid biosynthesis pathway. This investigation establishes a theoretical framework for the production of superior, bioactive finger millet sprout ingredients.

## 1. Introduction

Flavonoids are a class of natural nutrients widely distributed in plants [1], serving as a key indicator in studies of plant stress physiology and functional nutrition [2]. Furthermore, flavonoids serve as potent natural antioxidants, exhibiting anti-inflammatory, antibacterial, antiviral, and anticancer activities [3,4], Thus, flavonoids confer substantial health benefits to humans. Accumulating evidence indicates that these compounds exhibit diverse physiological protective activities, potentially reducing the incidence of metabolic syndrome and various malignancies [5]. Wang [6] further confirmed that flavonoid consumption is demonstrably correlated with a diminished likelihood of atherosclerotic occurrence. Therefore, the development of functional foods enriched in flavonoids holds considerable research significance.

Finger millet (*Eleusine coracana* L.), a monocarpic cereal of the *Panicoideae* subfamily, is abundant in bioactive compounds, including flavonoids and phenolic acids [7], establishing it as a prime precursor for the fabrication of flavonoid-enriched functional foods. Moderate abiotic stress effectively activates plant secondary metabolism, thereby increasing the content of functional components, such as flavonoids. Zinc sulfate (ZnSO_4_), a widely used zinc salt, has been demonstrated to modulate secondary metabolism during plant germination and enhance flavonoid accumulation [8]. ZnSO_4_ treatment has been shown to effectively induce the activities of key enzymes in the phenylpropanoid pathway in plants such as chickpea [9], barley [10], and broccoli [11], thereby significantly enhancing flavonoid accumulation. However, ZnSO_4_ stress, while promoting flavonoid accumulation, also elicits plant stress responses, resulting in adverse effects such as growth inhibition and oxidative damage. Our preliminary findings indicate that treatment with 5 mM ZnSO_4_ elicited flavonoid accumulation in finger millet sprouts while concurrently suppressing seedling elongation, fresh biomass accumulation, and chlorophyll content, thereby impeding growth and development [12]. Consequently, identifying safe and efficacious exogenous regulators that can mitigate the phytotoxic effects of zinc sulfate while promoting flavonoid enrichment is crucial for enhancing the quality of finger millet sprouts. Consequently, the identification of safe and effective exogenous regulators that can simultaneously enrich flavonoids and ameliorate ZnSO_4_-induced oxidative stress injury holds significant importance for enhancing the nutritional quality of finger millet sprouts.

The exogenous application of phytohormones constitutes a viable strategy to alleviate the suppressive impact of ZnSO_4_ stress on the growth and development of finger millet sprouts. Research has shown that the exogenous administration of specific compounds—including phytohormones such as salicylic acid (SA) [13], abscisic acid (ABA) [14], melatonin [15], and γ-aminobutyric acid (GABA) [16]—produces pronounced regulatory effects on plant physiological and metabolic pathways. They regulate the biosynthesis of secondary metabolites while simultaneously alleviating the inhibitory impacts of abiotic stress on plant growth.

SA is a widely distributed phenolic compound in plants and exerts multifaceted influences on the mechanisms underlying abiotic stress tolerance, including responses to heat, heavy metals, ozone, and osmotic stress [17]. Exogenous application of salicylic acid (SA) has been demonstrated to activate the phenylpropanoid metabolic pathway in wheat, upregulate the expression of flavonoid biosynthesis-associated genes, and consequently promote flavonoid accumulation. Moreover, this treatment enhances the plant’s antioxidant capacity, thereby mitigating oxidative damage induced by zinc stress [18]. GABA serves as a versatile biostimulant, significantly influencing plant responses to abiotic stress and facilitating the production of secondary metabolites. Recent investigations have revealed that applying GABA to the foliage of black wheat under conditions of low temperature and cadmium stress results in a reduction in malondialdehyde (MDA) and hydrogen peroxide (H_2_O_2_) concentrations. This treatment is also associated with a marked increase in the activities of superoxide dismutase (SOD) and peroxidase (POD) [19]. While prior studies have demonstrated that exogenous application of SA or GABA alleviates abiotic stress and promotes secondary metabolite accumulation, a systematic investigation of the physiological and metabolic responses—including effects on flavonoid biosynthesis—during the germination of finger millet sprouts under ZnSO_4_-induced stress, when each compound is applied individually, remains lacking.

This study investigated the effects of exogenous GABA and SA on physiological metabolism, the activities of key enzymes involved in flavonoid metabolism, antioxidant enzyme activities, and overall antioxidant capacity in finger millet sprouts under ZnSO_4_ treatment. Furthermore, the relative transcript abundance of genes implicated in flavonoid biosynthesis was quantified following exogenous application of SA and GABA under conditions of ZnSO_4_ treatment. These outcomes facilitate an enhanced comprehension of the mechanisms by which exogenous modulators influence physiological metabolic pathways and flavonoid accumulation within finger millet seedlings. The presented research outcomes offer a theoretical foundation for augmenting the quality of finger millet sprouts and advancing the development of plant-derived composite food products.

## 2. Results

### 2.1. Effects of Combined Exogenous Compounds on Flavonoid Content in Finger Millet Sprouts Under ZnSO_4_ Treatment

**Figure 1:**
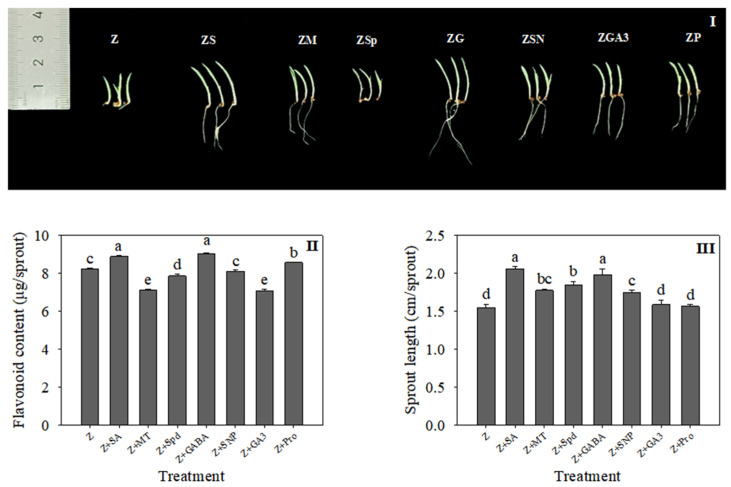
Effects of combined exogenous substances and ZnSO_4_ treatments on flavonoid content and sprout length in Finger Millet (*Eleusine coracana* L.) Sprouts. Note: Morphological images of finger millet sprouts under varied treatments (**I**), sprout flavonoid content (**II**), sprout length (**III**). Z: 5 mM ZnSO_4_; Z + SA: 5 mM ZnSO_4_ + 100 μM SA; Z + MT: 5 mM ZnSO_4_ + 0.1 mM MT; Z + Spd: 5 mM ZnSO_4_ + 0.5 mM Spd; Z + GABA: 5 mM ZnSO_4_ + 1.2 mM GABA; Z + SNP: 5 mM ZnSO_4_ + 0.1 mM SNP; Z + GA3: 5 mM ZnSO_4_ + 1 mM GA3; Z + Pro: 5 mM ZnSO_4_ + 10 mM Pro. Lowercase characters denote statistically significant differences among treatments within the same germination period, as determined by Tukey’s Honestly Significant Difference (HSD) post hoc analysis (*p* < 0.05).

As illustrated in Figure 1, after 6 days of germination, the combined application of various exogenous compounds with ZnSO_4_ markedly influenced both flavonoid content and sprout length in finger millet sprouts (*p* < 0.05). Relative to ZnSO_4_ treatment alone, the ZS and ZG treatments produced a significant elevation in flavonoid accumulation and promoted sprout growth, while other exogenous substances exhibited limited enhancement effects. Overall, SA and GABA were identified as the optimal exogenous compounds for synergistically improving both flavonoid content and seedling growth when combined with ZnSO_4_; therefore, these two additives were selected for subsequent experiments.

### 2.2. Optimization of Application Concentrations for Exogenous SA and GABA

**Figure 2:**
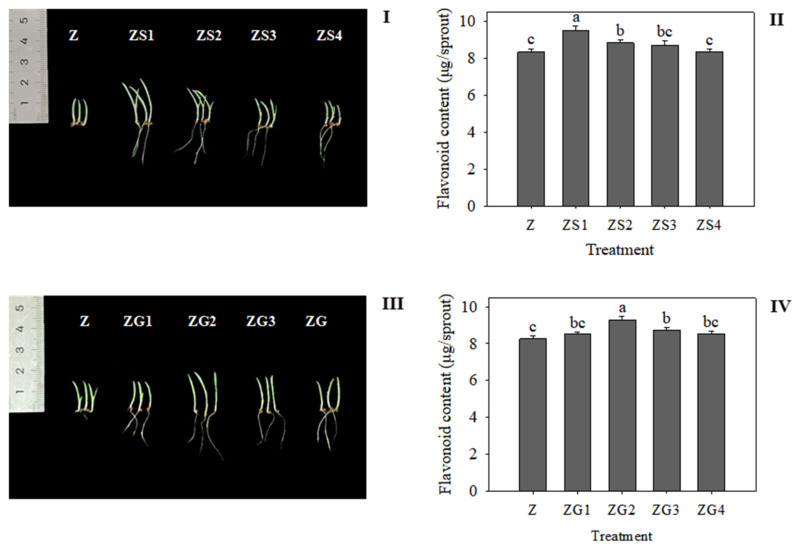
Optimization of exogenous SA and GABA concentrations for maximal efficacy. Note: Z: Morphology (**I**) and shoot length (**II**) under different SA concentrations; morphology (**III**) and shoot length (**IV**) under different GABA concentrations; 5 mM ZnSO_4_; ZS1: 5 mM ZnSO_4_ + 50 μM SA; ZS2: 5 mM ZnSO_4_ + 100 μM SA; ZS3: 5 mM ZnSO_4_ + 150 μM SA; ZS4: 5 mM ZnSO_4_ + 200 μM SA; ZG1: 5 mM ZnSO_4_ + 0.5 mM GABA; ZG2: 5 mM ZnSO_4_ + 1 mM GABA; ZG3: 5 mM ZnSO_4_ + 1.5 mM GABA; ZG4: 5 mM ZnSO_4_ + 2 mM GABA. Lowercase characters denote statistically significant differences among treatments within the same germination period, as determined by Tukey’s Honestly Significant Difference (HSD) post hoc analysis (*p* < 0.05).

To identify the optimal concentrations for combined SA and GABA treatments with ZnSO_4_, a concentration gradient screening experiment was performed. Results indicated that exogenous application of SA markedly enhanced the growth morphology of finger millet sprouts compared to ZnSO_4_ treatment alone. Specifically, treatment with 50 μM SA yielded the highest flavonoid content, significantly surpassing other concentrations and reaching 9.43 μg per sprout (Figure 2I,II). Furthermore, combined application of exogenous GABA and ZnSO_4_ improved morphogenesis relative to ZnSO_4_ alone, with the 0.5 mM GABA combination producing optimal sprout growth and an 18.24% increase in flavonoid content compared to ZnSO_4_ treatment alone (Figure 2III,IV). The findings demonstrate that treatment with 50 μM SA and 1.0 mM GABA significantly enhances sprout morphology and promotes the accumulation of nutritional constituents, thereby establishing these concentrations as optimal for future experimental investigations.

### 2.3. Effects of Exogenous SA/GABA on Flavonoid, Total Phenolic, and GABA Content in Finger Millet Sprouts Under ZnSO_4_ Treatment

At day 6 of germination, the flavonoid content in finger millet sprouts subjected to both ZS and ZG treatments reached its maximum. Under the ZS treatment, total phenolic content was significantly increased by 10.65% and 10.36% at days 4 and 6 of germination, respectively, compared to the ZnSO_4_ alone treatment (*p* < 0.05). The GABA content in germinated finger millet sprouts subjected to ZG treatment exhibited a continuous increase with prolonged germination, reaching its maximum value on day 6 of germination (Table 1). These results indicate that, under ZnSO_4_ treatment, exogenous application of SA and GABA can enhance the flavonoid and total phenolic contents in finger millet sprouts during germination. Furthermore, exogenous GABA may exert a positive regulatory effect on the biosynthesis of endogenous GABA in the plant.

### 2.4. Effects of Exogenous SA/GABA on $O_{2}^{-•}$, H_2_O_2_ and MDA Levels in Finger Millet Sprouts Under ZnSO_4_ Treatment

**Figure 3:**
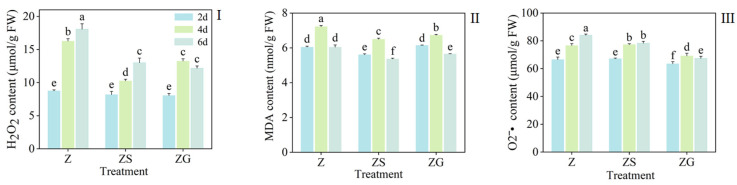
Effect of exogenous SA/GABA on H_2_O_2_ (**I**), MDA (**II**), and $O_{2}^{-•}$ (**III**) content of finger millet sprouts under ZnSO_4_ treatment; 2 d: 2 days post-germination; 4 d: 4 days post-germination; 6 d: 6 days post-germination. Lowercase characters denote statistically significant disparities among treatment cohorts at discrete germination time points, as elucidated by Tukey’s Honestly Significant Difference (HSD) post hoc analysis (*p* < 0.05).

Exposure to ZnSO_4_ stress elicited elevated levels of reactive oxygen species (ROS) in finger millet sprouts, culminating in oxidative injury. To elucidate the modulatory effects of exogenous SA and GABA on this response, oxidative stress markers were assessed, with results presented in Figure 3. Compared with ZnSO_4_ treatment alone, both ZS and ZG treatments led to a significant reduction in H_2_O_2_ content in finger millet sprouts (*p* < 0.05). Specifically, H_2_O_2_ content in the ZS treatment group decreased by 37.00% and 28.00% on days 4 and 6 of germination, respectively. In addition, on day 6 of germination, $O_{2}^{-•}$ content was significantly reduced in both ZS and ZG groups, with the ZG group exhibiting a 19.28% decrease. Notably, MDA content was also significantly reduced in both ZS and ZG treatments on days 4 and 6 of germination.

In summary, exogenous application of SA and GABA effectively alleviated oxidative damage induced by ZnSO_4_ stress in finger millet sprouts.

### 2.5. Impact of Exogenous SA/GABA Application on the Antioxidant Enzyme Profile and Overall Antioxidant Potential of Finger Millet Seedlings Undergoing ZnSO_4_ Exposure

**Figure 4:**
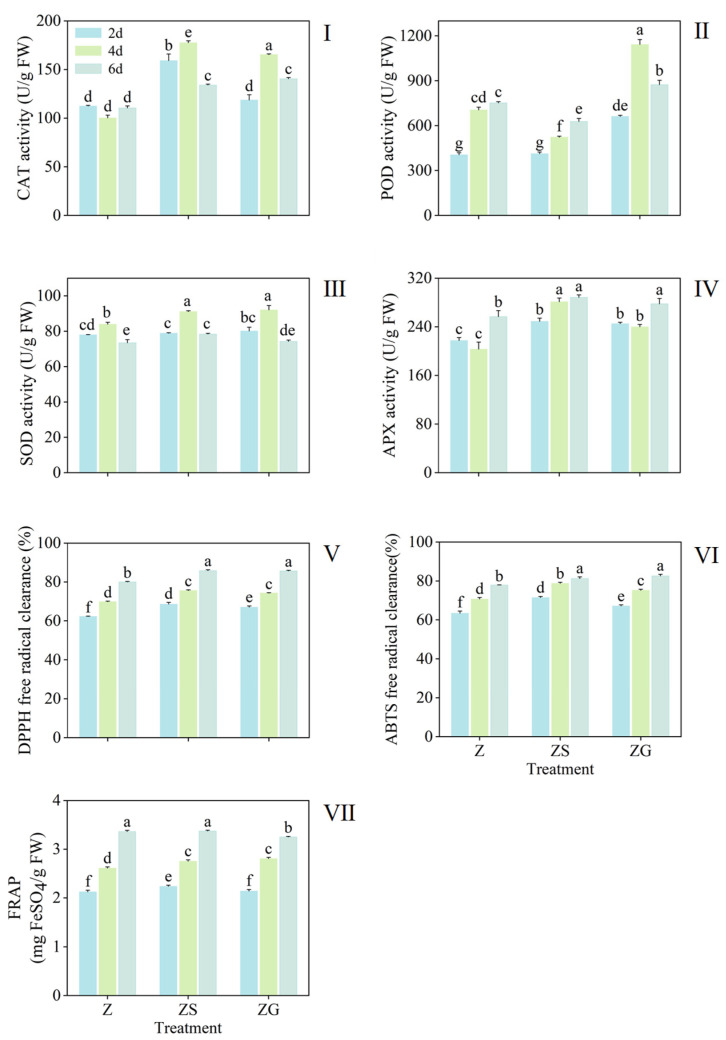
Effect of exogenous SA/GABA on Antioxidant enzyme activities and antioxidant capacities of finger millet sprout under ZnSO_4_ treatment. CAT activity (**I**), POD activity (**II**), SOD activity (**III**), APX activity (**IV**), DPPH free radical clearance (**V**), ABTS free radical clearance (**VI**) and FRAP (**VII**); 2 d: 2 days post-germination; 4 d: 4 days post-germination; 6 d: 6 days post-germination. Lowercase characters denote statistically significant disparities among treatment cohorts at discrete germination time points, as elucidated by Tukey’s Honestly Significant Difference (HSD) post hoc analysis (*p* < 0.05).

Compared with ZnSO_4_ treatment alone, the combined exogenous application of SA and GABA with ZnSO_4_ significantly modulated the activities of antioxidant enzymes (CAT, POD, SOD, and APX) in finger millet sprouts (*p* < 0.05) (Figure 4I–IV). Both ZS and ZG treatments enhanced CAT activity, with ZG treatment increasing CAT activity by 64% and 27% at 4 and 6 days of germination, respectively, relative to ZnSO_4_ alone. For POD activity, ZS treatment resulted in a decrease, whereas ZG treatment led to a significant increase compared to ZnSO_4_ alone. SOD activity was significantly elevated by ZS treatment at both 4 and 6 days of germination, while ZG treatment showed a significant increase only at day 4 of germination. In addition, both ZS and ZG treatments markedly increased APX activity throughout germination, with respective increases of 18.20% and 38% at day 4 of germination compared to ZnSO_4_ treatment.

DPPH, ABTS, and FRAP are core indices for assessing the antioxidant capacity of plants. Figure 4V–VII indicate that exogenous SA/GABA significantly enhances the antioxidant capacity of finger millet sprouts under ZnSO_4_ stress. Compared to ZnSO_4_ treatment alone, both ZS and ZG treatments exhibited maximal DPPH radical scavenging rates at 6 days of germination (Figure 4V). Regarding ABTS radical scavenging activity, the ZS treatment resulted in increases of 12.69%, 11.31%, and 6.45% at 2, 4, and 6 days of germination, respectively, while the ZG treatment showed relatively stable improvements of 6.56%, 7.12%, and 6.85% at 2, 4, and 6 days of germination, respectively (Figure 4VI). FRAP analysis demonstrated that ZS treatment significantly enhanced ferric reducing ability at 2 and 4 days of germination, whereas the effect of ZG treatment was only evident on day 4 (Figure 4VII). Collectively, these results demonstrate that exogenous SA and GABA effectively increase DPPH and ABTS radical scavenging rates, as well as FRAP activity, in finger millet sprouts under ZnSO_4_ treatment, with a general trend of increasing efficacy over time. This indicates a significant modulatory effect of SA and GABA on the antioxidant system of finger millet sprouts subjected to ZnSO_4_ stress.

### 2.6. Effects of Exogenous SA/GABA on Flavonoid Synthase Activity in Finger Millet Sprouts Under ZnSO_4_ Treatment

**Figure 5:**
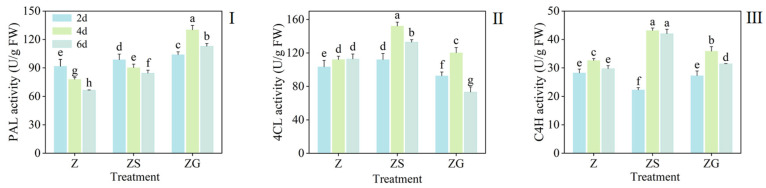
Effect of exogenous SA/GABA on PAL (**I**), 4CL (**II**), and C4H (**III**) activity of finger millet sprout under ZnSO_4_ treatment; 2 d: 2 days post-germination; 4 d: 4 days post-germination; 6 d: 6 days post-germination. Lowercase characters denote statistically significant disparities among treatment cohorts at discrete germination time points, as elucidated by Tukey’s Hon-estly Significant Difference (HSD) post hoc analysis (*p* < 0.05).

This study evaluated the impact of SA and GABA on the activities of these enzymes in finger millet sprouts under ZnSO_4_-induced stress. As shown in Figure 5, results demonstrated that, compared to ZnSO_4_ treatment alone, the ZG treatment led to the most significant activation of PAL, with increases of 66.88% and 69.79% on days 4 and 6 of germination, respectively, both significantly exceeding those observed in the ZS group (*p* < 0.05). ZS treatment significantly increased 4CL activity by 15.38% and 27.06% on days 4 and 6 of germination, reaching a maximum at day 4 of germination, while 4CL activity in the ZG group decreased by day 6 of germination. In the ZS group, C4H activity showed a continuous increase, rising by 32.35% and 41.34% on days 4 and 6 of germination, respectively, whereas ZG treatment significantly enhanced C4H activity only on day 4 of germination by 10.09%.

Collectively, these data indicate that exogenous SA and GABA attenuate ZnSO_4_-induced stress in finger millet sprouts through upregulation of key phenylpropanoid pathway enzymes, yet they exert their effects via distinct regulatory mechanisms. GABA predominantly enhances PAL activity, whereas SA preferentially upregulates the activities of C4H and 4CL.

### 2.7. Exogenous SA/GABA Regulates Flavonoid Metabolism and Antioxidant Gene Expression in Finger Millet Sprouts Under ZnSO_4_ Treatment

**Figure 6:**
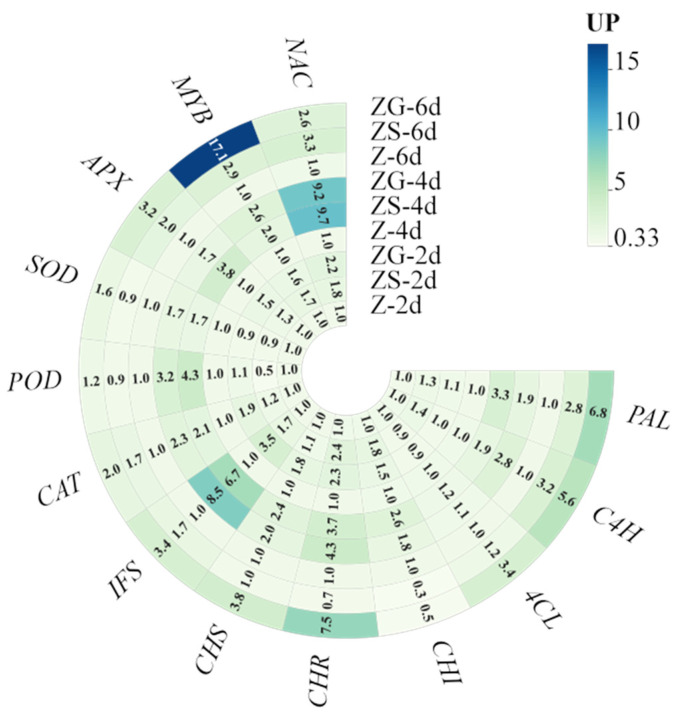
Influence of exogenous SA and GABA on the transcriptional profiles of genes associated with finger millet sprout development under ZnSO_4_ exposure. Z-2 d/4 d/6 d: ZnSO_4_ alone at day 2, 4, 6; ZS-2 d/4 d/6 d: ZnSO_4_ + SA; ZG-2 d/4 d/6 d: ZnSO_4_ + GABA. Genes include antioxidant enzymes (APX, SOD, POD, CAT), flavonoid biosynthetic enzymes (PAL, C4H, 4CL, CHI, CHR, CHS, IFS), and transcription factors (MYB, NAC).

Exogenous application of SA and GABA in combination with ZnSO_4_ significantly upregulated the expression of key genes involved in phenylpropanoid and flavonoid biosynthetic pathways (*EcPAL*, *EcC4H*, *Ec4CL*, *EcCHI*, *EcCHS*, *EcCHR*) in finger millet sprouts, as shown in Figure 6 Among these, the ZG treatment exhibited the most pronounced induction of *EcC4H*, *Ec4CL*, and *EcCHR*, with transcript levels at 6 days of germination reaching 5.6-fold, 3.4-fold, and 7.5-fold, respectively, compared to the ZnSO_4_ control. The ZS treatment significantly elevated the expression of *EcCHI*, *EcCHS*, and *EcCHR* at 4 days of germination, with transcript abundance 2.6-fold, 2.4-fold, and 3.7-fold higher, respectively, than the ZnSO_4_ treatment. Furthermore, combined application of SA, GABA, and ZnSO_4_ markedly enhanced the expression of antioxidant enzyme genes (*EcCAT*, *EcPOD*, *EcSOD*, *EcAPX*) and transcription factor genes (*EcMYB*, *EcNAC*). The expression of *EcPOD* peaked at day 4 of germination, with ZS and ZG treatments resulting in levels 4.3-fold and 3.2-fold higher, respectively, than the ZnSO_4_ group. ZG treatment significantly activated *EcAPX*, *EcMYB*, and *EcNAC*, with *EcAPX* transcript abundance at day 6 reaching 3.2-fold that of the ZnSO_4_ treatment, *EcMYB* at day 6 reaching 17.1-fold, and *EcNAC* at day 4 reaching 9.7-fold and 9.2-fold, respectively, relative to ZnSO_4_. These findings indicate that exogenous SA and GABA can effectively activate flavonoid metabolism and the antioxidant system.

## 3. Discussion

In response to abiotic stresses such as salinity, drought, and heavy metals like ZnSO_4_, numerous exogenous compounds, including SA and GABA, have been demonstrated to enhance plant stress tolerance. Acting as key plant signaling molecules, these substances bolster resistance by modulating physiological metabolism, regulating signaling pathways, and promoting the biosynthesis of specialized metabolites [20,21]. Although the exogenous application of SA or GABA alone can induce the flavonoid biosynthetic pathway and produce a modest increase in flavonoid accumulation [17,19], exclusive reliance on such single elicitors fails to generate stress-specific reactive oxygen species (ROS) signals. This deficiency results in insufficient activation of the plant’s global antioxidant and secondary metabolic regulatory networks [22]. As a consequence, the magnitude of flavonoid enhancement attainable through the individual application of SA or GABA is subject to a distinct saturation threshold. Low-concentration ZnSO_4_ induces ROS signal generation [23], forming signal crosstalk with the SA and GABA hormone pathways, which further upregulates the transcript abundance of flavonoid biosynthesis-related genes and enhances enzyme activity, ultimately leading to efficient flavonoid accumulation under stress conditions. Furthermore, as an essential trace element for humans, moderate zinc enrichment in germinated grains does not introduce food safety risks; instead, it enhances the nutritional value of the product [24,25]. In this study, we evaluated the effects of varying concentrations of SA and GABA on flavonoid accumulation in finger millet sprouts subjected to ZnSO_4_ treatment. Our results indicate that supplementation with 50 μM SA and 1.0 mM GABA significantly elevated levels of flavonoids were observed in finger millet sprouts. In addition, these treatments improved key physiological parameters, including morphology, sprout length, and fresh weight. These findings suggest that exogenous application of SA and GABA mitigates ZnSO_4_-induced stress in finger millet sprouts, alleviating growth inhibition while promoting the accumulation of flavonoids and other secondary metabolites.

Abiotic stress can impede normal plant development and suppress various physiological processes, frequently leading to the generation of ROS within the cells. As detrimental byproducts of plant stress metabolism, excessive accumulation of ROS results in membrane lipid peroxidation, thereby damaging cellular membrane integrity. MDA, H_2_O_2_, and $O_{2}^{-•}$ levels are key indicators for assessing the extent of cellular damage. This study found that, compared to treatment with ZnSO_4_ alone, the addition of exogenous SA and GABA significantly reduced MDA and H_2_O_2_ levels in finger millet sprouts at both 4 and 6 days of germination, as well as $O_{2}^{-•}$ levels at 6 days of germination. Similarly, Liu et al. [26] reported that exogenous application of SA reduced the levels of MDA and $O_{2}^{-•}$ in soybean roots, while foliar application of 5 mM GABA by Wang et al. [19]. Alleviated cadmium-induced growth inhibition in ryegrass seedlings and decreased the concentrations of MDA and H_2_O_2_. The observed regulatory effects align with findings from prior investigations employing combined treatments of finger millet sprouts with SA/GABA and ZnSO_4_. It is well established that abiotic stress induces both catalytic and non-catalytic antioxidative protective mechanisms [27,28]; key antioxidant enzymes such as CAT, POD, SOD, and APX are essential constituents of the plant antioxidant system, efficiently mitigating oxidative stress caused by ROS accumulation and thereby preserving redox homeostasis [29]. In this study, compared to the ZnSO_4_ treatment group, the combined application of exogenous SA and GABA with ZnSO_4_ markedly enhanced the activities of CAT, POD and APX in finger millet sprouts at both 4 and 6 days of germination, while SOD activity was only elevated in sprouts at 4 days of germination. SOD activity was enhanced only in the 4-day-old sprouts. Comparative analysis revealed that different antioxidant enzymes exhibit distinct temporal patterns in response to zinc sulfate stress: SOD is activated solely during early germination, whereas the other antioxidant enzymes undergo sustained induction. Phased gene transcription is a potential cause of this differential response. Furthermore, the addition of SA/GABA increased the relative transcript levels of *EcCAT* and *EcAPX* at 4 and 6 days of germination, mirroring the observed trends in enzymatic activity. Exogenous SA mitigates cadmium-induced adverse effects in rapeseed via enhancement of endogenous antioxidant defense systems, as evidenced by increased activities and relative transcript levels of SOD, POD, APX, and CAT enzymes in both leaves and roots [30]. Exogenous SA stimulates antioxidant and glutathione metabolism, inducing the transcription of related genes, thereby mitigating oxidative stress and enhancing alfalfa’s tolerance to zinc stress [31]. Additionally, GABA modulates the transcription and activity of specific enzymes in peanut sprouts under UV-C radiation, leading to increased antioxidant enzyme activities and elevated expression of isoenzymes, which in turn alleviates UV-C-induced oxidative stress in peanut sprouts [32]. Exogenous SA and GABA ameliorate the deleterious consequences of ZnSO_4_ stress in finger millet sprouts by enhancing the activities and transcriptional levels of CAT, POD, and APX, thereby facilitating the removal of excess ROS.

Transcription factors (MYB and NAC) play crucial roles in regulating plant responses to abiotic stress conditions [33,34]. Numerous studies have investigated the involvement of transcription factors in hormone-mediated abiotic stress responses [35,36]. However, the response of transcription factors to exogenous GABA during plant stress adaptation remains unclear. In particular, the roles of *EcMYB* and *EcNAC* under ZnSO_4_ stress in the context of GABA regulation have yet to be explored. The investigation revealed that SA significantly augmented *EcMYB* relative expression in finger millet sprouts at 6 days post-germination. SA also substantially elevated *EcNAC* relative expression in sprouts at both 4 and 6 days post-germination. Comparable findings were reported by Zheng et al. [37], who demonstrated that foliar application of SA upregulated MYB gene expression in *Dianthus superbus* L., thereby improving the plant’s tolerance to moderate salt stress (0.3% and 0.6% NaCl). This suggests that under severe abiotic stress, protein damage may occur, thereby suppressing the expression of genes such as MYB and inhibiting enzyme biosynthesis. The combined application of GABA and ZnSO_4_ significantly enhanced the expression levels of *EcMYB* in finger millet sprouts at both 4 and 6 days of germination. This finding further indicates that GABA induced the upregulation of *EcMYB*, which may be one of the mechanisms underlying the improved tolerance of finger millet sprouts to ZnSO_4_ stress. Studies have demonstrated that the overexpression of NAC genes can significantly enhance plant tolerance to abiotic stress [38]. In the present study, both SA and GABA were found to markedly induce *EcNAC* expression in 4-day- and 6-day-old finger millet. Transcription factors, specifically those belonging to the MYB, NAC, and basic leucine zipper (bZIP) families, are recurrently identified as key regulators of plant responses to abiotic stressors, primarily by enhancing the biosynthesis of secondary metabolites [39]. This finding further substantiates the increase in flavonoid and total phenolic contents in finger millet sprouts under combined SA and GABA with ZnSO_4_ stress.

The biosynthesis of flavonoids in plants primarily proceeds via the phenylpropanoid and flavonoid metabolic pathways and is tightly regulated by numerous enzymatic activities and gene expression. To investigate the mechanistic roles of exogenous SA and GABA in flavonoid biosynthesis under ZnSO_4_ treatment, we analyzed the activities and gene expression levels of three key enzymes: PAL, C4H and 4CL. The results indicate that SA significantly increased the activities of PAL, C4H, and 4CL in finger millet sprouts germinated under ZnSO_4_ stress for 4 and 6 days. This phenomenon is also consistent with the findings of Niu et al. [40]; these effects promoted the synthesis of secondary metabolites and mitigated stress-induced damage. However, the expression level of *Ec4CL* in finger millet sprouts treated with the combined application of SA and ZnSO_4_ did not increase compared to those treated with ZnSO_4_ alone. Co-treatment with GABA and ZnSO_4_ significantly enhanced the activities of PAL and C4H in sprouts at 4 days of germination, and also upregulated the transcript levels of *EcPAL* and *EcC4H* at the same time point. Similarly, Wang et al. [41] reported that exogenous GABA application markedly increased both the activities and mRNA expression of PAL, C4H, and 4CL under low NaCl conditions in barley sprouts. These findings indicate that exogenous SA or GABA may promote flavonoid accumulation in finger millet sprouts by upregulating key enzymes and their corresponding genes in the phenylpropanoid pathway.

The flavonoid metabolic pathways, constituting the downstream segment of flavone biosynthesis, are critically shaped by the action of metabolic enzyme genes such as *EcCHI*, *EcCHS*, *EcCHR*, and *EcIFS*. Research by Huang et al. [42] demonstrated a positive correlation between the relative expression levels of *GmCHI1A*, *GmCHS*, *GmCHR*, and *GmIFS1* and the accumulation of flavones in soybean. In this study, SA enhanced the relative expression levels of *EcCHI*, *EcCHR*, and *EcCHS* in 4-day-old germinating finger millet sprouts subjected to ZnSO_4_ treatment. However, this finding does not align with the observation that flavonoid content peaks at 6 days of germination, suggesting that this discrepancy may be attributable to the spatiotemporal variability in gene expression [43]. *EcNAC* displayed a canonical early stress response profile, with transcript abundance at 4 days post-germination exceeding that at 6 days. GABA treatment consistently upregulated *EcMYB* expression at both sampling intervals. As upstream regulators within stress signal transduction cascades, these transcription factors coordinately initiate the transcription of multiple downstream defense genes during the initial stress phase. At 4 days post-germination, zinc stress signaling reached peak intensity, coinciding with the co-activation of flavonoid biosynthesis genes (*EcCHI*, *EcCHS*) and the SOD-encoding gene, resulting in abundant SOD protein accumulation. This observation accounts for the significant elevation of SOD activity exclusively at the 4-day time point. By 6 days, finger millet sprouts had progressively acclimated to zinc sulfate stress, accompanied by a decline in stress-related transcriptional activity and reduced SOD protein synthesis. In contrast, *EcCAT* and *EcAPX* maintained stable expression throughout the experimental period, leading to sustained increases in CAT and APX activities at both 4 and 6 days. Collectively, these findings indicate that spatiotemporal specificity of gene expression constitutes the core molecular mechanism underlying temporal differentiation of various physiological indices under stress conditions. The relative expression level of *EcCHI* in the combined GABA and ZnSO_4_ treatment followed a pattern similar to that observed in the SA plus ZnSO_4_ group, peaking at day 4 of germination but displaying a marked decrease by day 6 compared to ZnSO_4_ treatment alone. As a key gene involved in substrate recognition during flavonoid biosynthesis, *EcIFS* expression in sprouts at day 6 was significantly upregulated by the addition of exogenous SA or GABA under ZnSO_4_ treatment. These findings indicate that co-application of SA or GABA with ZnSO_4_ enhances flavonoid biosynthesis via transcriptional upregulation of genes located further down the flavonoid metabolic pathway.

## 4. Materials and Methods

### 4.1. Experimental Design

Finger millet was procured in 2024 from the agricultural market of Zayü County, Tibet Autonomous Region. Following sterilization, experimental seeds were rinsed 3–5 times with deionized water until neutral pH was achieved and then imbibed in deionized water at 29 °C for 6 h, after which they were evenly distributed onto germination trays for sprouting. This investigation commenced with an exogenous substance screening utilizing a 5 mM ZnSO_4_ treatment, thereby constructing composite treatment cohorts comprising seven exogenous substances: 100 μM SA, 0.1 mM melatonin (MT), 0.5 mM spermidine (Spd), 1.2 mM GABA, 0.1 mM sodium nitroprusside (SNP), 1 mM gibberellic acid 3 (GA_3_), and 10 mM proline (Pro). After 6 days of germination, SA and GABA were identified as candidates due to their observed enhancement of flavonoid biosynthesis. Subsequently, concentration optimization experiments were conducted for SA and GABA. Concentration gradients were established for SA (25, 50, 100, 200 μM) and GABA (0.5, 1, 1.2, 2 mM). After 6 days of germination, the optimal exogenous treatment conditions were determined to be 50 μM SA and 1 mM GABA, based on sprout flavonoid content and growth parameters. The experimental design comprised three treatment groups: a control group receiving only 5 mM ZnSO_4_; a ZS treatment group (5 mM ZnSO_4_ + 50 μM SA); and a ZG treatment group (5 mM ZnSO_4_ + 1 mM GABA). The treatment solutions were applied via spraying every 12 h during germination, with a total volume of 150 mL per tray per application. Samples were collected for analysis on days 2, 4, and 6 of germination. Finger millet sprouts exhibiting uniformity in height, growth vigor, and morphology, and free from physical damage, were selected during sampling. Each biological replicate comprised a composite of thirty finger millet sprouts. All pooled samples were collected, their initial fresh weights recorded, and subsequently ground for extraction and determination of subsequent physiological indicators.

### 4.2. Determination of Flavonoid, Total Phenolic, and GABA Content

Determination of flavonoid content was conducted according to the method described by Vélez et al. [44]. Briefly, 3 g of finger millet sprouts was homogenized in 6 mL of 80% (*v*/*v*) ethanol and subjected to sonication for 25 min at 30 °C using an ultrasonic cleaner (Shenzhen Jiemai Ultrasonic Cleaning Equipment Co., Ltd., Shenzhen, China). Following centrifugation using an HC-2066 high-speed centrifuge (Anhui USTC Zonkia Scientific Instruments Co., Ltd., Hefei, China), the supernatant was collected. Absorbance was measured at a wavelength of 510 nm on a UV-1150 spectrophotometer (Shanghai Mapada Instruments Co., Ltd., Shanghai, China) using rutin as the reference standard, with results expressed as μg/g fresh weight (FW).

Total phenolic content was quantified utilizing the established methodology of Wang et al. [45]. Phenolic compounds were extracted using methanol. Absorbance measurements were performed at 765 nm, with gallic acid employed as the calibration standard, using the gallic acid standard curve for reference, and reported as mg GAE/g FW.

GABA content was quantified following the established methodology of Zhao et al. [46]. A total of 0.6 g of finger millet sprouts was homogenized with 5 mL of ultrapure water. The homogenate was subjected to ultrasonic extraction at 60 °C for 35 min. Following centrifugation, the supernatant was collected. GABA content was quantified using a modified Berthelot colorimetric assay, and the results were expressed as mg/g fresh weight (FW).

### 4.3. Determination of MDA, H_2_O_2_, and Superoxide Anion Radical ($O_{2}^{-•}$) Content

MDA content was quantified utilizing the protocol previously delineated by Yin et al. [47]. The thiobarbituric acid (TBA) colorimetric assay was utilized for quantification.

The H_2_O_2_ and $O_{2}^{-•}$ content was quantified according to the methodology described by Zhao et al. [48]. Finger millet sprouts (0.5 g) were homogenized using phosphate buffer. The resultant homogenate was centrifuged at 8000× *g* for 5 min under low-temperature conditions, yielding a supernatant for analysis. H_2_O_2_ content was quantified using the xylenol orange colorimetric method, with absorbance measured at 560 nm. $O_{2}^{-•}$ content was quantified via the hydroxylamine hydrochloride colorimetric assay, 20 min incubation at room temperature was performed after homogenizing the extract with the detection reagent; an additional 20 min reaction was conducted upon the addition of a color-developing solution containing 17 mM α-naphthylamine, followed by centrifugation at 1500× *g* for 5 min. Absorbance was subsequently measured at 530 nm.

### 4.4. Quantification of Antioxidant Enzyme Activity

SOD activity was quantified utilizing the methodology previously detailed by Azevedo et al. [49]. POD and catalase (CAT) activities were quantified following the methodology outlined by Wang et al. [50].

### 4.5. Assessment of Antioxidant Capacity

The antioxidant capacity of the samples was quantified via three established assays, adhering to the specific operational protocols detailed by Rumpf et al. [51]. DPPH radical scavenging activity assay: Sample solutions were mixed with DPPH ethanolic solution and incubated in the absence of light. Absorbance was subsequently measured at 517 nm. Ferric reducing antioxidant power (FRAP) assay: Sample solutions were mixed with freshly prepared FRAP working solution and incubated in a water bath at 37 °C. Absorbance was measured at 593 nm, with results expressed as equivalents of Fe^2+^. ABTS radical scavenging activity assay: Sample solutions were mixed with the ABTS working solution activated with potassium persulfate, sealed, and incubated at room temperature until the reaction reached equilibrium. Absorbance was then measured at 734 nm.

### 4.6. Determination of Flavonoid Metabolic Enzyme Activities

PAL, C4H, and 4CL enzyme activities were quantified following the established protocol of Wang et al. [52]. Specifically, 1.5 g of finger millet tissue was pulverized and homogenized in ice-cold Tris-HCl buffer. The resulting homogenate underwent low-temperature, high-speed centrifugation. The supernatant, representing the crude enzyme extract, was subsequently harvested. Enzyme activity assays were conducted utilizing the reaction parameters, termination procedures, and absorbance measurement wavelengths as delineated in the referenced methodology. All quantified activities were reported as units per gram of fresh weight (U/g FW).

### 4.7. RNA Extraction and Real-Time Quantitative PCR Analysis

The finger millet samples were ground into a fine powder in liquid nitrogen and subsequently transferred to centrifuge tubes. Lysis buffer was added to ensure thorough homogenization and complete cell disruption. Total RNA was extracted following the manufacturer’s instructions using the TAKARA Plant RNA Extraction Kit (Catalog No.: 9769). The extracted RNA was reverse transcribed into cDNA using the RT Kit (TAKARA, RR036A, Shiga, Japan) following the manufacturer’s protocol. The resulting cDNA was stored at −80 °C for future analysis. In this study, we utilized the complete genomic sequence data of finger millet available in the NCBI database and referenced gene primer sequences reported in the relevant literature [53] to design specific primers using Primer 5.0 software. The primer sequences for the reference gene (Actin) and the target genes are listed in Table S1. Quantitative real-time PCR (qRT-PCR) was performed using the TB Green Premix DimerEraser kit (RR091A, TAKARA, Japan) according to the manufacturer’s instructions. Relative expression levels of key flavonoid metabolism enzyme genes in finger millet sprouts were analyzed against the reference gene (Actin), and gene expression was quantified using the 2^−ΔΔCt^ method [54]. A PCR reaction system was prepared using the TB Green Premix DimerEraser kit (Cat. No. RR091A, TAKARA, Shiga, Japan) according to the manufacturer’s instructions, and the corresponding PCR amplification conditions were set.

### 4.8. Statistical Analysis

All experimental data pertinent to this investigation were derived from independent triplicates, with resultant data presented as mean ± standard deviation. Statistical analyses were conducted utilizing the DPS data processing system (version 9.50). One-way analysis of variance (ANOVA) was employed for inter-group difference testing, followed by Tukey’s method for multiple comparisons of means. Significance was determined at the 0.05 level (*p* < 0.05).

## 5. Conclusions

Exogenous application of SA (50 μM) or GABA (1 mM) in combination with ZnSO_4_ significantly reduced the levels of MDA, H_2_O_2_, and $O_{2}^{-•}$ in finger millet sprouts, thereby mitigating oxidative damage and alleviating growth inhibition. Furthermore, SA combined with ZnSO_4_ enhanced both the activities and gene expression of CAT, APX, and SOD; while GABA combined with ZnSO_4_ elevated the activities and gene expression of CAT, POD, and APX, indicating that both exogenous treatments bolstered antioxidative capacity. Exogenous SA or GABA also facilitated flavonoid accumulation in finger millet sprouts by increasing the activities of key flavonoid biosynthetic enzymes (PAL and C4H) and upregulating the expression of related anabolic genes. The foregoing results indicate that the combined application of SA or GABA with ZnSO_4_ serves as an effective regulatory measure, significantly enhancing the functional quality of finger millet sprouts.

## Figures and Tables

**Table 1:** Effects of exogenous SA/GABA on flavonoid, total phenolic, and GABA contents in finger millet sprouts under ZnSO_4_ treatment.

Treatment		Flavonoid Content (μg/g FW)	Total Phenolic Content (μg GAE/g FW)	GABA Content (mg/100 g FW)
Z	2 d	349.00 ± 3.51 ^c^	449.88 ± 14.08 ^g^	47.49 ± 1.36 ^e^
	4 d	381.76 ± 16.46 ^c^	491.43 ± 8.43 ^e^	52.73 ± 2.66 ^de^
	6 d	424.25 ± 26.15 ^c^	582.70 ± 5.14 ^c^	62.04 ± 2.35 ^c^
ZS	2 d	423.83 ± 29.18 ^c^	484.11 ± 7.34 ^ef^	56.15 ± 2.15 ^cd^
	4 d	527.78 ± 16.97 ^b^	543.79 ± 9.93 ^d^	63.29 ± 1.68 ^c^
	6 d	565.64 ± 32.71 ^ab^	643.10 ± 7.40 ^a^	53.63 ± 4.12 ^de^
ZG	2 d	507.50 ± 57.70 ^b^	466.69 ± 3.33 ^fg^	57.10 ± 1.26 ^cd^
	4 d	569.43 ± 26,019 ^ab^	522.05 ± 7.48 ^d^	72.00 ± 3.23 ^b^
	6 d	646.42 ± 11.16 ^a^	614.13 ± 8.40 ^b^	83.06 ± 2.62 ^a^

Note: Mean values within a column sharing dissimilar superscript lowercase letters signify statistically significant disparities among treatment groups (*p* < 0.05). These distinctions were ascertained via one-way analysis of variance (ANOVA) coupled with Tukey’s Honestly Significant Difference (HSD) post hoc analysis; 2 d: 2 days post-germination; 4 d: 4 days post-germination; 6 d: 6 days post-germination.

## Data Availability

The original contributions presented in this study are included in the article/Supplementary Materials. Further inquiries can be directed to the corresponding author.

## Supplementary Materials

The following supporting information can be downloaded at: https://www.mdpi.com/article/10.3390/plants15132065/s1, Table S1: The sequences of the primers utilized in the study.
