# Supplementary material for: Effects of Exogenous SA/GABA Combined with ZnSO4 Treatment on the Physiological Metabolism and Flavonoid Biosynthesis in Finger Millet (Eleusine coracana L.) Sprouts
Source: Plants (Basel). 2026 Jul 2;15(13):2065. doi: 10.3390/plants15132065 (PMC13363764; doi:10.3390/plants15132065)
Supplement: Supplementary file 1 [file plants-15-02065-s001.zip › plants-4384263-supplementary.pdf]

Table S1 The sequences of the primers utilized in the study

Name	Forward Primer (5' – 3')	Reverse Primer (5' – 3')
Actin	CTCACGCTCAAGTACCCCAATC	GGCAACACGAAGCTCATTGTAC
EcCAT	ACCCGCCTTTACCTACCTTTTT	CATACGCCGAAAAGCATCCAT
EcSOD	CTCCTACCGGCGACCTCTACCCAGC	CTGAGGCTTGTCCTCCCTCCCTG
EcPOD	CCAGGTGCTCTACCTCCGACGACC	GAGGTTGGTCATGGCGGCGAC
EcAPX	CACCTGTTCCTCGACTTTGC	TTACCGTTGCAGCAGTTGAGG
EcPAL	CGTGCCGCTCTCCTACCATTGC	CCTCTGCTGCGTTCACCTTGG
EcC4H	GACTTCCGCTTCCTGCCGTTC	CACGAGCTTGCCGACGATGAG
Ec4CL	GACGACAAGGCGACCAAGGC	CTCCACGCTGCTGATGTTCTCG
EcCHI	GCCGCCGTGGAGAAGTTCAAG	ACCGACGAGTCCTTGGAGAACG
EcCHR	AGTCTCAAGATCGCATTGCTGGTG	AACTTGTGGTGAGGTGTGCTGTG
EcCHS	ATGCTGTTCTCCGTCCCGAATTTC	CTTACTCTTCCTGGCGAGCACCTTC
EcIFS	AAGCAAGCGGATGTGGTGTTCTC	GCTCCACGTCACAGCCATATTCAG
EcIFR	CCTGGCTCGCCGTCAACAAG	GGATGCTGCTGGCTCTGCTG
EcNAC	CGTGTGCAAGGTGTTCAACA	CCAAGTAGTCGCTGAAGGAG
EcMYB	AGGAGGAGGAAGATGCTGAAAGT	TTGAGGTGGTTGGATACGTGAGAG

Note: CAT: catalase; SOD: superoxide dismutase; POD: peroxidase; APX: ascorbate peroxidase; PAL: phenylalanine ammonia-lyase; C4H: cinnamate 4-hydroxylase; 4CL: 4-coumarate: coenzyme A ligase; CHI: chalcone isomerase; CHR: chalcone reductase; CHS: chalcone synthase; IFR: isoflavone reductase ； IFS: isoflavone synthase; C4H: cinnamate 4-hydroxylase
